# Prevalence of dyslexia related to mental health problems and character strengths among primary school students in northwest China

**DOI:** 10.1080/00049530.2024.2399114

**Published:** 2024-09-09

**Authors:** Weiyan Feng, Rassamee Chotipanvithayakul, Hongyu Liu

**Affiliations:** a Department of Epidemiology, Faculty of Medicine, Prince of Songkla University, Hat Yai, Thailand; b Research Center for Child and Youth Development, Faculty of Medicine, Prince of Songkla University, Hat Yai, Thailand; c School of Humanities and Management, Ningxia Medical University, Yinchuan, China

**Keywords:** Dyslexia, bullying, self-esteem, depression, anxiety, character strengths

## Abstract

**Objective:**

This study aimed to examine the prevalence of dyslexia, mental health problems, and character strengths among primary school students in northwest China.

**Method:**

Primary school students (*N* = 2,322) were assessed for dyslexia, strengths and difficulties, self-esteem, bullying, depression, anxiety, and character strengths. The Student’s t-test and χ^2^ test were used to compare continuous and categorical variables between students with and without dyslexia.

**Results:**

The estimated prevalence of dyslexia ranged from 4.9% to 6.9% and was highly prevalent among boys. Students with dyslexia reported statistically higher mean scores for emotional symptoms, conduct behaviours, internalizing and externalising problems, as well as total difficulties. Conversely, they exhibited lower levels of prosocial behaviour, expressed lower self-esteem, and reported higher instances of being bullied. Additionally, the prevalences of depression and anxiety symptoms were 36.5% and 26.3%, respectively, among dyslexic students. Signature strengths among students with dyslexia included appreciation of beauty and excellence, love, hope, forgiveness, and judgement.

**Conclusions:**

These findings underscore dyslexia’s impact on mental health and academic performance in China. Implementing character strength-focused interventions could enhance well-being and academic outcomes among dyslexic primary school students.

## Key Points

**What is already known about this topic:**

1. The global prevalence of dyslexia was recognised; however, research in northwest China is overlooked.
2. Dyslexia impacted on both mental health and academic progress among children.
3. Enhance character strengths could foster resilience and well-being.

**What this topic adds:**

1. This study addressed the under-recognition of dyslexia by reporting a 5.9% dyslexia prevalence among primary school students in Yinchuan city, northwest China.
2. The mental health and character strengths of primary school students were comprehensively explored, which revealed higher difficulties and mental health problems but also unique strengths in dyslexic students.
3. Integrated mental health interventions are advocated by emphasising early dyslexia identification and recommending character strength promotion for all students to realise the potential benefits of academic and mental well-being in all students, especially young students with dyslexia.

## Introduction

Dyslexia is a neurodevelopmental disorder that impacts reading, writing, and spelling. It is acknowledged as a specific learning disability (Lyon et al., 2003). Despite its global prevalence, ranging from 5% to 17.5%, dyslexia has predominantly been studied in Western contexts, with an estimated prevalence of 7.1% among primary students worldwide (Hinshelwood, 1900; Shaywitz, 1998; Yang et al., 2022). However, the unique structures, educational methodologies, and cultural contexts of English and Chinese educational systems highlight the need for region-specific research.

In China, several epidemiological surveys in the southern regions have reported various prevalence rates of dyslexia, with Guangzhou, Hong Kong, Qianjiang, and Shantou showing rates of 5.4%, 9.7%, 7.0% and 5.4%, respectively, and a rate of 3.9% in Xinjiang, which is in the northwest region (Cai et al., 2020; Chan et al., 2007; Lin et al., 2020; Sun et al., 2013; Zhao et al., 2016). Despite its prevalence in China, research on dyslexia is still limited. In numerous regions of China, including northwest China, dyslexia remains largely unrecognised by parents, teachers, and even the Ministry of Education. There is a lack of established screening or intervention programmes for students with dyslexia. Furthermore, the implications of dyslexia on the mental health of primary school students in the northwest region have not been adequately addressed.

Recent studies have highlighted the heightened vulnerability of dyslexic students to mental health challenges that include elevated emotional symptoms, conduct issues, and peer interaction difficulties (Jordan & Dyer, 2017; Karami et al., 2019; Knivsberg & Andreassen, 2008; Oei et al., 2016; Terras et al., 2009). These challenges often manifest in diminished self-esteem, increased risks of internalising and externalising problems, depression, and anxiety (Terras et al., 2009; Xiao et al., 2022). Their susceptibility to bullying further exacerbates these issues, thereby impacting both overall well-being and academic progress (Crick & Bigbee, 1998; Hawker & Boulton, 2000; Mishna, 2003; Ridsdale et al., 2005; Singer, 2005). Furthermore, even if children with dyslexia are not formally identified, they still experience stigma, which can adversely affect their mental health and academic performance. Research indicates that individuals with dyslexia are prone to self-stigmatisation and may use derogatory terms such as “stupid” or “unintelligent” to describe themselves. A systematic review conducted in 2023 demonstrated that higher levels of dyslexia-related stigma are associated with poorer outcomes, including increased anxiety and depression, as well as decreased academic performance, adaptive coping, self-efficacy, and quality of life (Haft et al., 2023).

Character strengths, as defined within positive psychology, encompass attributes that influence cognitive, emotional, and behavioural facets that play a pivotal role in fostering resilience and well-being (Peterson & Seligman, 2004). Some researchers reported that intervention to promote signature strengths could increase happiness and life satisfaction (Proyer et al., 2015; Schutte & Malouff, 2019). Several studies listed in Table 1 reported character strengths that were associated with reduced conduct problems and hyperactivity, depression, anxiety, increased self-esteem, bullying, and school achievement. However, there is a paucity of literature on how Chinese primary school students with dyslexia recognise and utilise their own strengths. Chinese culture, with its long history and profound moral values, significantly influences the development of children. Confucianism, a distinctly Chinese philosophical tradition, emphasises virtues such as wisdom, humanity, and righteousness, which play a pivotal role in shaping the conception of character strengths (Yan et al., 2022).

**Table 1.** Character strengths related to mental health in previous studies.

Mental health	Character strengths	Mental health	Character strengths
Conduct problems and hyperactivity (Qin et al., 2022)	Hope	Depression (Proyer et al., 2015)	Hope
	Self-regulation		Zest
Anxiety (Qin et al., 2022)	Appreciation of beauty and excellence		Self-regulation
	Love	School Achievement (Wagner & Ruch, 2015)	Perseverance
	Perseverance		Self-regulation
	Social intelligence		Hope
	Zest		Honesty
	Perspective		Creativity
	Creativity		Prudence
Self-esteem (Toback et al., 2016)	Signature strengths		Zest
Bullying (Gülbahar & Sarı, 2022)	Humility		Social intelligence
	Honesty		Love of learning
	Perseverance		Perspective

Moreover, studies have shown a useful and logical cumulative risk-resilience model of dyslexia. The model indicates that whereas multiple risk factors, such as phonological deficits, language impairments, attentional deficits, visual problems, and trauma/stress, increase the likelihood of severe and persistent difficulties in learning to read, resilience (positive) factors can reduce this likelihood. This theoretical model supports character strengths as the protective and resilience factors to reduce the impact of risk factors among dyslexic children (Catts & Petscher, 2022; Haft et al., 2016). Thus, interventions focusing on character strengths have emerged as promising for enhancing the well-being of dyslexic students.

Despite the evident challenges, dyslexia remains under-recognised in northwest China, which has led to inadequate psychological and academic support for affected students. Our study aimed to address this gap by (1) determining the prevalence of dyslexia among primary school students in Yinchuan city in northwest China, (2) assessing the strengths, difficulties, and mental health of dyslexic students, and (3) identifying character strengths in dyslexic Chinese students for further development of positive psychology interventions.

By illuminating the challenges and strengths of dyslexic students in northwest China, this study aspired to advance academic understanding and guide targeted interventions for this distinct demographic profile.

## Methodology

### Study setting and participants

This school-based survey was conducted in public primary schools in Yinchuan city, Ningxia Hui Autonomous Region in northwest China in 2023. The selection of schools was based on gross domestic product, which included 203 primary schools in all six districts that were classified into low, moderate, and high socio-economic levels. Nine schools were randomly chosen with proportional probability sampling to size from both rural and urban areas in each socio-economic level. Subsequently, one classroom from each of five grades (grades 2–6) was randomly selected in each school. A total of 2,225 students was required to estimate a 7% dyslexia prevalence rate that accounted for a 2.5% precision and a 10% refusal rate. Students with a history of brain injury, visual and auditory disorders, epilepsy, and other neurological disorders were excluded.

### Measures

Questionnaires, which were completed by the students and their parents, were used to gather background information and assess mental health. After collecting all questionnaires, dyslexic students were screened by the Chinese language teachers.

#### Mental health

Six standard questionnaires in Chinese versions were used to assess mental health: (1) Strengths and Difficulties Questionnaire-Parent version (SDQ-P); (2) the Self-Esteem Scale for Children (SESC); (3) the Delaware Bullying Victimisation Scale (DBVS); (4) the Children’s Depression Inventory (CDI); (5) the Screen for Child Anxiety Related Emotional Disorders (SCARED); and (6) the VIA Youth. The mean times to complete questionnaires ranged from 20–45 minutes.

##### Strengths and Difficulties Questionnaire-Parent version (SDQ-P)

The SDQ-P (Lai et al., 2010), which assessed the students’ behaviour problems, was completed by the parents. It is a reliable test with a test-retest reliability value of 0.80, content validity of 0.80, and a Cronbach’s α coefficient of 0.3–0.77. The SDQ-P is comprised of 25 items distributed across 5 subscales where each is scored from 0 to 10: (1) conduct problems (0–2: normal, 3: borderline, 4–10: abnormal); (2) emotional symptoms (0–3: normal, 4: borderline, 5–10: abnormal); (3) peer problems (0–2: normal, 3: borderline, 4–10: abnormal); (4) hyperactivity-inattention (0–5: normal, 6: borderline, 7–10: abnormal); and (5) prosocial behaviour (6–10: normal, 5: borderline, 0–4: abnormal). The ranges of the total difficulties score, which excluded prosocial behaviour, were 0–13 (normal), 14–16 (borderline), and 17–40 (abnormal) (Goodman, 1997).

##### The Self-Esteem Scale for Children (SESC)

The SESC (Wei, 1997) was used to assess the self-esteem of students aged 6–12 years. The SESC consisted of 26 items that measured 6 subscales: appearance; sports; ability; sense of achievement; discipline; and public morality and helpfulness, which demonstrated a Cronbach’s alpha value of 0.68 for reliability. The score of each item ranged from 1–5. Higher scores indicated a higher self-esteem level.

##### The Delaware Bullying Victimization Scale (DBVS)

The DBVS (Fang et al., 2021) is a self-response questionnaire that measures exposure to bullying. It is comprised of 12 items divided into three dimensions: physical bullying (4 items); verbal bullying (4 items); and social bullying (4 items). The scores range from 0–5 that indicate the level of bullying experienced by the student from never to everyday exposure to serious bullying. It has a Cronbach’s α reliability of 0.8 and a reliability of 0.7 (Xie et al., 2015).

##### The Children’s Depression Inventory (CDI)

The CDI (Kovacs, 2015) was used to assess the severity of depressive symptoms. The CDI has 27 items with scores from 0 (no symptom), to 2 (definite symptom). The total score is 54 points and the cut-off point for the symptom of depression is 20 (Matthey & Petrovski, 2002). Cronbach’s alpha values of the CDI ranged from 0.70 to 0.87.

##### The Screen for Child Anxiety Related Emotional Disorders (SCARED)

The SCARED questionnaire (Linyan et al., 2008) was used to assess panic anxiety, generalised anxiety, separation anxiety, social anxiety, and significant school avoidance. Each item is scored from 0 (no symptom) to 2 (frequent symptoms). Scores higher than 30 suggest anxiety disorder. Scores higher than 7, 9, 8, and 3 suggest panic disorder, generalised anxiety disorder, separation anxiety disorder, social anxiety disorder, and school avoidance anxiety, respectively. It has moderate to high levels of internal consistency (α = 0.4–0.9) and excellent distinctiveness validity between anxious and normal groups.

##### The VIA Youth

The VIA Youth (Miller et al., 2021) has 103 items in 24 subscales. The VIA Youth was used to assess 24 character strengths among students aged 8–12 years. The internal consistency was between 0.6 and 0.9.

#### Dyslexia screening

All eligible students who underwent dyslexia screening were assessed by the Combined Raven’s Test (CRT) for intelligence quotient (IQ). The CRT has been widely used for IQ screening in Chinese children. The test-retest stability of the CRT was 0.97 and the validity was 0.66 (Li et al., 1988). Students who scored below 75, which indicates borderline intellectual functioning, were excluded. Additionally, the Chinese language teacher used the Pupil Rating Scale-Revised (PRS) (Jing et al., 1995) to assess learning disabilities in students whose Chinese language test scores fell within the lowest 20% of their class in the final exam. The PRS evaluated five domains: auditory comprehension and memory; language; time and orientation judgement; motor; and social behaviour. It exhibited high reliability (test-retest coefficient > 0.80) and moderate validity coefficients that ranged from 0.5 to 0.6. Any student scoring below 65 on this assessment was also excluded (Cai et al., 2020). Subsequently, the remaining students were subjected to further evaluation using the Dyslexia Checklist for Chinese Children (DCCC) (Hou et al., 2018), which was completed by the Chinese language teacher. Students who scored 2 standard deviations (SD) higher than the mean score in the same grade were classified as having dyslexia. The test demonstrated excellent reliability (α = 0.97) and validity.

### Procedure

Approvals to conduct the study were received from the school principals. Comprehensive information about the study was provided to the teachers, students, and parents. Written informed consent was obtained from both the students and parents. Researchers provided formal training at each school to the Chinese language teachers on how to use these assessment tools. The researchers were present with the teachers to provide support and supervision. The voluntary nature of participation was emphasised, and the students had the right to refuse or discontinue at any time. Researchers distributed a set of self-response questionnaires that included the Combined Raven’s Test, SESC, DBVS, CDI, SCARED, and the VIA Youth scales to each student in their classroom. The researchers attended the class to ensure that the students answered the questionnaires independently, and assistance was provided as needed. Students had two 10-minute breaks to relax. Students put the completed questionnaires into an envelope and handed it to the researcher. Additionally, the Chinese language teachers were asked to screen for dyslexia using the PRS and DCCC scales. The researchers subsequently collected them within one week. The background data and SDQ-P questionnaires were given to the parents. After completing the questionnaires, the parents put them in an envelope, and the students returned the envelope to the teacher the following day or within a week. Subsequently, the researcher collected the questionnaires from the teacher.

### Ethics statement

The study was approved by the Institutional Ethics Committee of the Faculty of Medicine, Prince of Songkla University, Thailand (65-331-18-9), and Ningxia Medical University, China.

### Data analysis

Data were entered into EpiData 3.1 (Lauritsen et al., 2003) and validated. Data cleaning and analysis were performed using R software version 4.3.2 (Chongsuvivatwong, 2012). Descriptive statistics were used to estimate the prevalence of dyslexia and mental health status. The Student’s t-test and χ^2^ test were used to compare continuous and categorical variables between dyslexia and non-dyslexia groups, respectively. Multiple t-tests and χ^2^ tests can inflate the family-wise error rate (FWER). To accurately adjust for the FWER, we applied the Bonferroni correction separately within each category based on the number of comparisons made in that category. The Bonferroni correction was chosen to control for the increased risk of Type I errors due to multiple comparisons. This adjustment ensures that the significance level is appropriately maintained.

## Results

**Figure 1.**
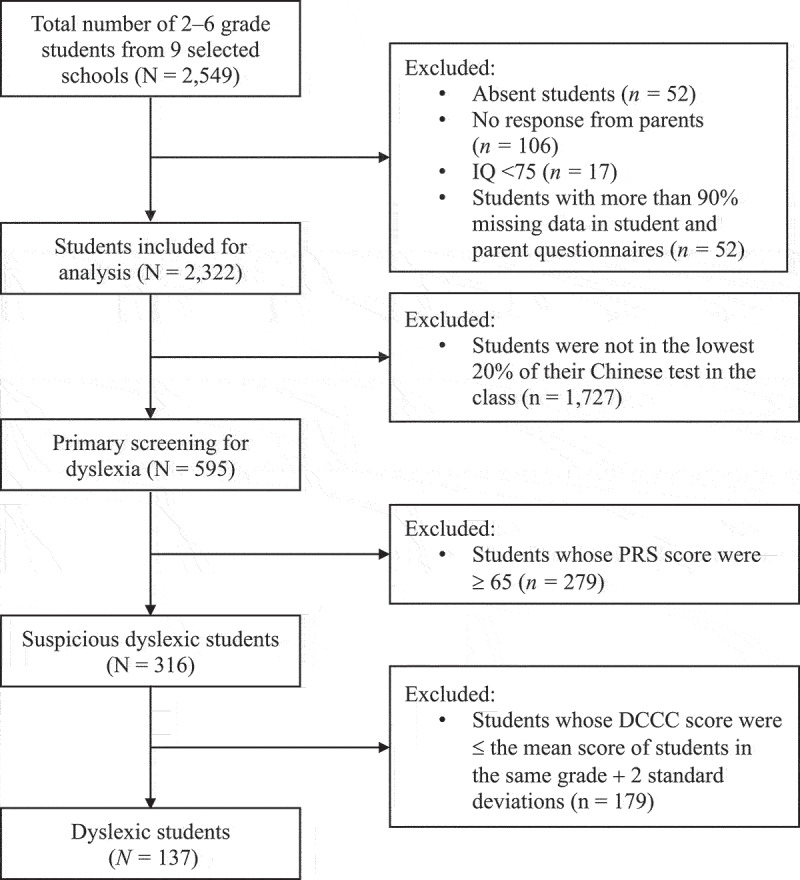
Screening dyslexia flowchart. IQ, intelligence quotient; PRS, pupil rating scale-revised screening for learning disability; DCCC, dyslexia checklist for Chinese children.

Out of the 2,549 primary school students from grades 2–6 and their parents who were invited to participate in the study across nine selected schools, 52 students were absent, and 106 parents refused to participate (95.7% response rate). Seventeen students were excluded due to an IQ lower than 75. Additionally, 52 students were excluded due to more than 90% of missing data in a set of self-response questionnaires and questionnaires completed by the parents. After these exclusions, the final analysis included a cohort of 2,322 students (Figure 1).

### Prevalence of dyslexia among primary school students

Out of 2,322 students, the remaining 1,727 students were excluded from dyslexia screening because they were not in the lowest 20% of their Chinese test in the class, and 279 students scored 65 or higher on the PRS screening for learning disability. Eventually, 316 students were eligible for dyslexia screening. One hundred and thirty-seven students (5.9%, 95% confidence interval [CI]: 4.9–6.9%) were identified as dyslexia by the DCCC test. The highest prevalence rate of dyslexia was observed in the second grade, and dyslexia tended to decrease in the higher grades without statistical significance (*p* = 0.427). The prevalence of dyslexia was more common among male than female students (Table 2).

**Table 2.** Prevalence of dyslexia by grades and gender among primary school students (*N* = 2,322).

	Dyslexia (*n* = 137)	Non-dyslexia (*n* = 2,185)	Prevalence Rate (%)
**Gender**			
Female	38	1,109	3.3
Male	99	1,076	8.4
**Grade**			
2nd	32	439	6.8
3rd	30	469	6.0
4th	31	471	6.2
5th	26	376	6.5
6th	18	430	4.0
Total	137	2,185	5.9

### Socio-demographic characteristics and birth history between dyslexic and non-dyslexic primary school students

Table 3 shows that students with dyslexia compared with students without dyslexia were significantly more likely to be male, have lower father’s education, and lower household monthly income. Additionally, we did not observe statistical differences in birth weight and length, history of premature birth, delivery mode, having siblings, or age of the parents.

**Table 3.** Socio-demographic characteristics between primary school students with and without dyslexia (*N* = 2,322).

	Dyslexia *n* = 137	Non-dyslexia *n* = 2,185	*p*-value
**Gender**			<.001
Female	38 (27.7)	1,109 (50.8)	
Male	99 (72.3)	1,076 (49.2)	
**School grade**			.427
2nd	32 (23.4)	439 (20.1)	
3rd	30 (21.9)	469 (21.5)	
4th	31 (22.6)	471 (21.6)	
5th	26 (19.0)	376 (17.2)	
6th	18 (13.1)	430 (19.7)	
**Maternal education level**			.074
Junior high school or below	89 (65.0)	1,226 (56.1)	
Senior high school or junior college or equivalency	28 (20.4)	477 (21.8)	
Junior college or above	20 (14.6)	482 (22.1)	
**Paternal education level**			.015
Junior high school or below	84 (61.3)	1,178 (53.9)	
Senior high school or Junior college or equivalency	35 (25.5)	485 (22.2)	
Junior college or above	18 (13.2)	522 (23.9)	
**Occupation of mother**			.211
Farming, forestry, fishery worker	22 (16.1)	264 (12.1)	
Business or staff in institution/government*	39 (28.5)	753 (34.5)	
Others	76 (55.5)	1,168 (53.5)	
**Occupation of father**			.162
Farming, forestry, fishery worker	21 (15.3)	251 (11.5)	
Business or staff in institution/government*	51 (37.2)	977 (44.7)	
Others	65 (47.4)	957 (43.8)	
Household monthly income (RMB)^**^			<.001
≤5,000	92 (67.2)	1,087 (49.7)	
>5,000 to less than 15,000	38 (27.7)	962 (44.0)	
≥15,000	7 (5.1)	136 (6.2)	

*Including professional technical staff, principal of institution and government, office staff, service staff, business staff, production worker, transport worker, and other related occupations. **1 RMB ≅ 0.14 USD.

### Mental health between students with and without dyslexia

The SDQ-P scores were classified into normal, borderline, and abnormal groups. Table 4 shows that three of the five SDQ-P domains (i.e., hyperactive and inattention, emotional symptoms, and prosocial behaviours) were within normal limits. However, they had borderline conduct behaviours and abnormal peer relationship. A comparison between the two groups showed that students with dyslexia had statistically higher mean scores for emotional symptoms, conduct behaviours, externalising problems, total difficulties, and lower mean scores for prosocial behaviour. The results suggested higher difficulties and lower strengths among students with dyslexia than their peers.

**Table 4.** Psychological and mental health problems between primary school students with and without dyslexia (*N* = 2,322).

Psychological and mental health problems	Dyslexia *n* = 137 Mean (SD)	Non-dyslexia *n* = 2,185 Mean (SD)	*p*-value	BC** adjusted significance
Strength and difficulty problems by SDQ-P^*^				α = .006
Hyperactivity and inattention	4.4 (1.9)	4.3 (1.6)	.534	No
Emotional symptoms	2.8 (2.1)	2.1 (1.9)	<.001	Yes
Conduct behaviour	2.7 (1.7)	2.1 (1.3)	<.001	Yes
Peer relationship	4.0 (1.7)	4.2 (1.5)	.125	No
Prosocial behaviour	5.9 (2.4)	7.0 (2.0)	<.001	Yes
Internalizing symptoms	6.8 (3.2)	6.3 (2.7)	.037	No
Externalizing symptoms	7.1 (2.9)	6.4 (2.3)	<.001	Yes
Total difficulty	13.8 (5.4)	12.6 (4.2)	.002	Yes
**Self-esteem**				α = .007
Total self-esteem	86.2 (12.6)	93.4 (15.5)	<.001	Yes
Appearance	13.0 (4.0)	13.5 (4.0)	.216	No
Sports	9.7 (2.8)	10.3 (3.0)	.032	No
Ability	16.7 (4.8)	19.5 (5.8)	<.001	Yes
Sense of achievement	13.6 (3.4)	14.6 (3.3)	<.001	Yes
Discipline	13.5 (3.2)	15.2 (3.3)	<.001	Yes
Public morality and helpfulness	19.7 (3.6)	20.3 (3.7)	.031	No
**Exposure to bullying**				α = .013
Total bullying	10.8 (9.8)	8.0 (8.8)	<.001	Yes
Physical bullying	3.7 (3.8)	2.4 (3.1)	<.001	Yes
Verbal bullying	3.9 (4.0)	3.3 (3.7)	.059	No
Social bullying	3.1 (3.6)	2.3 (3.3)	.005	Yes
**Depression symptoms *n* (%)**	50 (36.5)	439 (20.1)	<.001	Yes (α = .05)
**Anxiety disorder symptoms *n* (%)**				α = .008
Total anxiety	36 (26.3)	439 (20.1)	.103	No
Panic	45 (32.8)	530 (24.3)	.031	No
Generalized anxiety	14 (10.2)	211 (9.7)	.947	No
Separation anxiety	60 (43.8)	786 (36.0)	.079	No
Social anxiety	22 (16.1)	315 (14.4)	.686	No
School avoidance anxiety	17 (12.4)	130 (5.9)	.005	Yes

*SDQ-P, Strengths and Difficulties Questionnaire-Parent version. **BC, Bonferroni correction.

Overall, students with dyslexia had lower self-esteem than their peers, exhibited lower levels of sense of achievement, perceived weaker abilities, and poorer discipline. Dyslexic students were more susceptible to physical and social bullying. They were also more prone to experiencing school avoidance anxiety·and depression.

**Figure 2.**
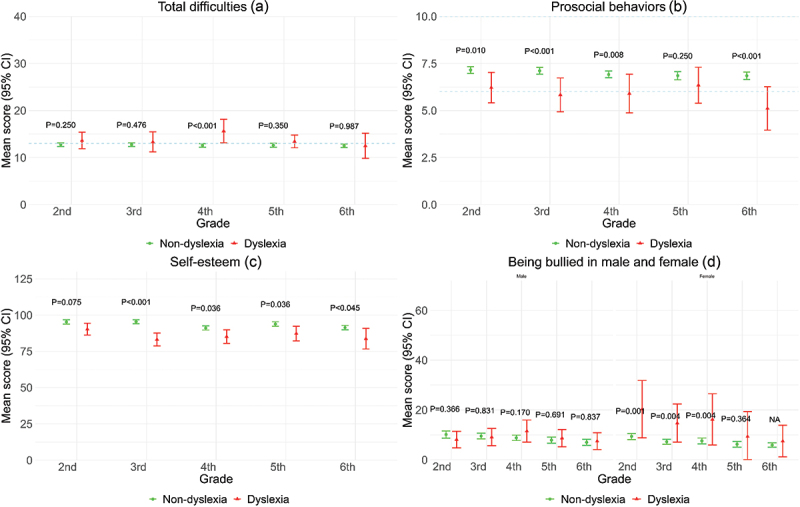
Comparison of SDQ-P scores, self-esteem, and being bullied by gender across school grades between non-dyslexic and dyslexic primary school students (*N* = 2,322). NA, *p*-value is not provided due to the small sample size.

Figure 2 illustrates a breakdown by school grades. The overall mean scores and 95% CI for total difficulties were generally low and comparable between students with dyslexia and their non-dyslexic counterparts (Figure 2a). However, students in the fourth grade with dyslexia displayed a significantly higher mean score than their peers. Conversely, students with dyslexia exhibited significantly lower mean scores in prosocial behaviours in all grades except students in the fifth grade (Figure 2b).

The average self-esteem scores of the students with dyslexia at the baseline measure in the second grade were minimally lower than the non-dyslexic students (Figure 2c). The lower self-esteem among students with dyslexia became more statistically significant over time. Although students with dyslexia had a significantly higher rate of bullying, we observed a statistical significance only among female students from the second through the fourth grades with significant interaction term (Figure 2d).

**Figure 3.**
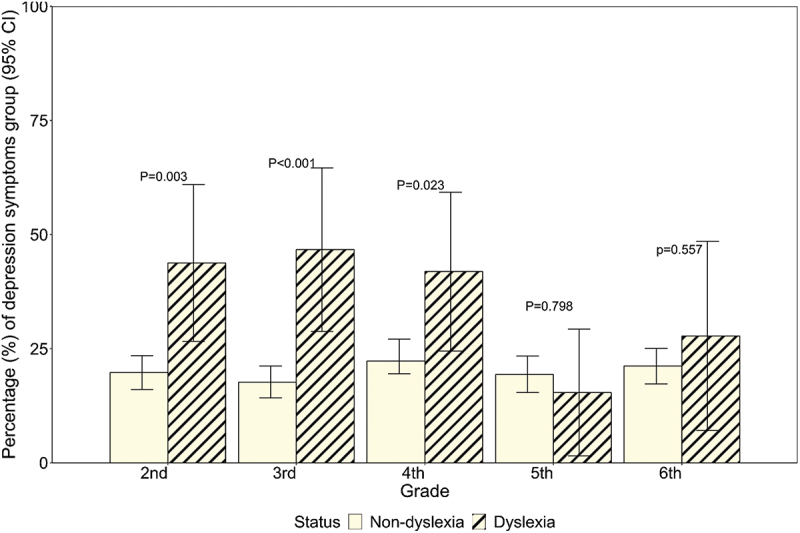
Comparison of depression symptoms by school grades between non-dyslexic and dyslexic primary school students (*N* = 2,322).

In grades 2–4, the percentages of depression symptoms among students with dyslexia were approximately twice as high as students without dyslexia (Figure 3).

### Character strengths between dyslexic and non-dyslexic students

Students with and without dyslexia shared common top three signature strengths that included appreciation of beauty and excellence, love, and hope. The last two signature strengths for dyslexic and non-dyslexic students were forgiveness and judgement, and judgement and love of learning, respectively. After applying the Bonferroni correction for significance, Table 5 shows that students with dyslexia had significantly lower scores in wisdom but higher scores in courage than the students without dyslexia. The scores were not statistically different in transcendence, humanity, temperance, and justice between the two groups. The rankings of virtues were also similar between them.

**Table 5.** Virtues between primary school students with and without dyslexia (*N* = 2,322).

Virtue	Dyslexia (*n* = 137) Mean (SD)	Rank	Non-dyslexia (*n* = 2,185) Mean (SD)	Rank	*p*-value
Transcendence	3.6 (0.6)	I	3.7 (0.6)	I	.009
Wisdom	3.5 (0.7)	II	3.7 (0.6)	II	<.001
Humanity	3.4 (0.6)	III	3.5 (0.5)	III	.132
Temperance	3.4 (0.6)	IV	3.4 (0.5)	IV	.893
Courage	3.2 (0.5)	V	3.1 (0.4)	VI	.006
Justice	3.2 (0.6)	VI	3.2 (0.5)	V	.456

**Figure 4.**
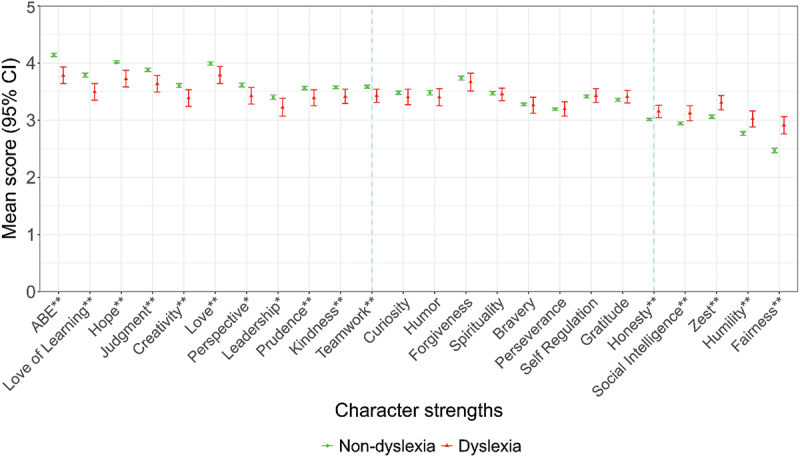
Comparison of character strengths between students with and without dyslexia (*N* = 2,322). ABE, appreciation of beauty and excellence. *p-values <0.05; **p-values <0.005.

Dyslexic students experienced more difficulties, increased risk of mental health problems, and were exposed to higher bullying. However, they had higher scores in some character strengths than their peers, which included honesty, social intelligence, zest, humility, and fairness (Figure 4). They had comparable scores with their peers for curiosity, humour, forgiveness, spirituality, bravery, perseverance, self-regulation, and gratitude. Dyslexic students had significantly lower scores than their non-dyslexic peers on the remaining character strengths, namely appreciation of beauty and excellence, love of learning, hope, judgement, creativity, love, perspective, leadership, prudence, kindness, and teamwork.

## Discussion

The current study reported a significant number of students with dyslexia among primary school students who not only had academic problems, but also had significant mental health issues.

### Prevalence of dyslexia among primary school students in northwest China

The prevalence of dyslexia was 5.9% (95% CI: 4.9–6.9%) among primary school students in northwest China. The highest rate was observed in the younger grades and gradually decreased in the higher levels. The prevalence in this study was similar to other Chinese studies in northwest China, for example Xinjiang (3.9%) and other parts including Guangzhou (5.4%), Shantou (5.4%) but lower than Qianjiang (7.0%) and Hongkong (9.7%). A previous meta-analysis in 2022 reported that the global prevalence rate of dyslexia among primary school students was 7.10% (95% CI: 6.27–7.97%) without differences between the high- and low-income countries (Yang et al., 2022). Dyslexia accounts for approximately 7.5 million and 55 million primary school students in China and worldwide, respectively. Dyslexia is a global health and education problem among children and requires special attention; however, dyslexia remains unrecognised in many students. Parents and teachers had very little knowledge of dyslexia in our setting and had no appropriate intervention for dyslexic students.

### Mental health problems

Compared to non-dyslexic students, those with dyslexia exhibited more difficulties and lower prosocial behaviours according to the results of the SDQ-P. Female students with dyslexia reported higher exposure to bullying that was possibly due to factors such as a reluctance to seek help, tendencies towards peer avoidance, and fears of negative peer comparisons (Mishna, 2003; Singer, 2005). Additionally, dyslexic students in general showed a higher prevalence of depression and low self-esteem, consistent with findings from studies conducted globally (Francis et al., 2019; Jordan & Dyer, 2017; Lun et al., 2008; Nalavany et al., 2018; Singer, 2008; Terras et al., 2009; Xu et al., 2020).

Despite having higher average scores for mental health issues compared to their peers, the dyslexic students in our study were within the normal range. Research indicated that mental health issues, including distress, depression, anxiety disorders, and suicidal ideation, increase significantly from adolescence and young adulthood (ages 15–21) to older adulthood (ages 30–44) among individuals with dyslexia (Wilson et al., 2009). This underscores primary school as a critical period for promoting mental health and preventing future disorders.

A multiple-component reading intervention for dyslexic students demonstrated efficacy in first-grade students who made substantial gains compared to second- and third-grade students (Lovett et al., 2017). Remediation of reading skills from the first grade onward improved a child’s self-esteem and mental health (Boyes et al., 2016).

This suggests that early diagnosis and treatment lead to better outcomes. Moreover, positive emotional support from teachers and parents enhances a child’s overall self-worth and fosters a positive attitude when facing reading difficulties while improving reading abilities and reducing anxiety (Terras et al., 2009). Clinicians and educators should consider both mental health and education issues (Moll et al., 2023).

Without timely appropriate intervention, students have increased risks of education problems that include grade repetition, truancy, school dropout (Aro et al., 2019; Lovett et al., 2017; Moll et al., 2023), and mental health problems. Furthermore, childhood emotional and behavioural disorders predicted various levels of criminality (Copeland et al., 2007). Another follow-up study also reported adults with dyslexia often obtained degrees post-compulsory education, experienced prolonged unemployment, and had relatively high rates of mental health issues (Aro et al., 2019).

### Character strengths

The top five signature strengths were similar between students with and without dyslexia. However, dyslexic students had significantly lower mean scores. The same four were appreciation of beauty and excellence, hope, love, and judgement. However, love of learning was the signature strength reported by only students without dyslexia and forgiveness was the strength reported by only students with dyslexia.

Enhancing the understanding and application of character strengths correlates significantly with elevated self-esteem (Govindji & Linley, 2007; Wood et al., 2011). Specific strengths such as creativity, zest, gratitude, and love exhibited dual influences on anxiety and depression with diminished levels correlating to heightened psychological distress (Bachik et al., 2020; Jabbari et al., 2021), which were common problems reported by students with dyslexia in our study. Based on signature strengths reported in this study and previous literature (Table 1), a programme that promotes appreciation of beauty and excellence, hope, love, judgement, and forgiveness could have benefits for the mental health of dyslexic students. Additionally, strengths such as prudence, perseverance, refined self-regulation, sound judgement, enriched perspective, enduring hope, love of learning, and the fostering of creativity should also be strengthened in all students, particularly those with dyslexia, to support academic achievement. These strengths can help students succeed academically, as demonstrated in programmes such as the Individualised Education Programme in the USA (Mueller & Vick, 2019).

According to the resilience concept and the cumulative risk-resilience model, our study suggests that character strengths can act as protective factors to reduce the risk of mental health issues and enhance academic performance among dyslexic students (Catts & Petscher, 2022; Haft et al., 2016).

In summary, the integration of universal programmes promoting character strengths can foster both mental well-being and academic achievement in all students, including those with dyslexia. Such programmes should be comprehensive and multidisciplinary, involving collaboration among students, parents, physicians, educators, psychologists, and social workers (Schulte-Körne, 2016). They should be initiated as early as the first grade and maintained at least through the sixth grade.

## Limitations

Several limitations should be acknowledged. Some of the young second-grade students, especially those with dyslexia, possibly had difficulties understanding and responding to the questionnaire that potentially led to random response bias. To mitigate this, researchers read the questions aloud and provided explanations. Also, the large sample size helped reduce the overall impact of random bias. Additionally, the study’s focus on a specific area of northwest China possibly limited the generalisability of the findings to other regions in China.

## Strengths

The study’s strengths include a large sample size, which enhanced the reliability and power of the findings. The use of random sampling reduced selection bias and ensured the representativeness of the findings in the study area. The application of appropriate tools and data analysis methods added rigour to the research. The inclusion of comparison groups allowed for robust conclusions. Importantly, the study not only identified the problems faced by students with dyslexia but also explored their strengths and resources that contributed to good internal validity.

## Conclusion

The prevalence of dyslexia (4.9–6.9%) among primary school students in Yinchuan city of northwest China was similar to other settings. Without any intervention, they are at a significantly increased risk of mental health and academic problems compared to their peers. Selected character strength-based intervention can be given to students with dyslexia to increase their good mental well-being and academic achievement without stigma.

## Data Availability

The data presented in this study are available upon reasonable request from the corresponding author. The data are not publicly available due to specific ethical and privacy considerations.
